# Challenges faced and lessons learned A multi-component prospective memory training program for Malaysian older adults

**DOI:** 10.1590/1980-57642018dn12-020012

**Published:** 2018

**Authors:** Azin Farzin, Rahimah Ibrahim, Zainal Madon, Hamidon Basri

**Affiliations:** 1 PhD. Postgraduate Student, Malaysian Research Institute on Aging, University Putra Malaysia (UPM), Serdang, Selangor, 43400, Malaysia.; 2 Research Associate, Malaysian Research Institute on Aging, University Putra Malaysia, Serdang, Selangor, 43400, Malaysia. Associate Professor, Department of Human Development and Family Studies, Faculty of Human Ecology, University Putra Malaysia, Serdang, Selangor, 43400, Malaysia.; University Putra Malaysia, Faculty of Human Ecology, Department of Human Development and Family Studies, Serdang, Selangor, Malaysia; 3 Senior Lecturer, Department of Human Development and Family Studies, Faculty of Human Ecology, University Putra Malaysia (UPM), Serdang, Selangor, 43400, Malaysia.; 4 Professor, Department of Medicine, Faculty of Medicine and Health Sciences, University Putra Malaysia (UPM), University Putra Malaysia (UPM), Serdang, Selangor, 43400, Malaysia.

**Keywords:** cross-over studies, aged, prospective memory, cognition disorders, activities of daily living, estudos cruzados, idosos, memória prospectiva, distúrbios cognitivos, atividades da vida diária

## Abstract

The surrounding circumstances and environments of Malaysian older adults could make conducting interventions (mainly in terms of clinical or randomized controlled trials) a challenge. Working with older adults and facing cultural issues could be challenging.

**Objective:**

This paper illustrates a significant perspective of some of the challenges faced while conducting a randomized controlled trial exploring the impact of a multi-component intervention that included strategy- and process-based prospective memory (PM) training among Malaysian older adults.

**Methods:**

The current study was a randomized controlled trial (RCT) and therefore the challenges were presented in accordance with the CONSORT statement style.

**Results:**

A discussion on how these issues were addressed is provided.

**Conclusion:**

Some suggestions were presented to help researchers plan and create interventions for similar studies and to support a practical method of addressing all related challenges.

## Resumo

As circunstâncias e ambientes circundantes de adultos idosos da Malásia poderiam tornar a condução da intervenção (principalmente em termos de ensaios clínicos ou randomizados controlados) um desafio. Trabalhar com adultos mais velhos e enfrentar problemas culturais pode ser instigante.

**Objetivo:**

Este artigo ilustra uma perspectiva significativa de alguns dos desafios enfrentados durante a realização de um estudo controlado randomizado que explora o impacto de uma intervenção de múltiplos componentes, incluindo treinamento de memória prospectiva baseado em estratégia e processo (PM) entre idosos da Malásia.

**Métodos:**

Como o estudo atual foi um ensaio clínico randomizado (ECR), os desafios foram apresentados de acordo com o estilo de declaração CONSORT.

**Resultados:**

Uma discussão sobre como essas questões foram abordadas é fornecida.

**Conclusão:**

Algumas sugestões foram apresentadas para ajudar os pesquisadores a planejar e criar intervenções para estudos semelhantes e capacitar um método prático para abordar todos os desafios relacionados.

Prospective memory (PM) literally means remembering to perform an ‘intended action’ at a certain point in the future.^1^ Some examples of PM functions include; remembering to water the plants, take medications, or attending an appointment. PM is frequently engaged in everyday life and is highly relevant for maintaining functional independence and wellbeing, especially among older adults.^2^^,^ 3 Ergo, the importance of studies which improve PM among older adults, especially in ´aging´ countries like Malaysia, is evident.^4^

However, conducting such studies might face several challenges. Moreover, lack of knowledge about aging and age-related changes, educational level and transportation can affect intervention results. Hence, the number of studies, in particular randomized controlled trials (RCTs) with similar approaches in such communities (e.g., Malaysian), is low.

Based upon the nature of the cue, PM can be divided into three types; time-based (e.g., remember to meet someone at 10:00 am), event-based (e.g., remember to mail a letter when passing the post office), and activity-based (e.g., remember to call a friend after lunch).^2^ Moreover, PM is a multi-step cognitive process functioning in four stages: [1] intention formation, [ii] the delay maintenance interval, [iii] self-initiated cue recognition and intention retrieval, and [iv] intention execution.^5^^,^ 6

PM is still, to some extent, a neglected research area. However, recent studies have shown that using computer-based training has a positive impact on memory and hence reduces the risk of Alzheimer’s disease (AD) among older adults.^7^ Some previous studies have used strategy-based and computer-based (laboratory-based) training to improve cognitive functions among older adults. Nevertheless, they failed to show any transfer effects to other everyday tasks.^8^^,^ 9 Other training studies have used process-based (computer-based) training to enhance PM performance among older adults. They also showed no real-life transfer effects from training.^10^ Several previous studies lacked methodological consistency and therefore conducting strictly controlled studies seems necessary. The randomized controlled trial (RCT) as an experimental design offers rigorous control for clinical trials and, based on the standards for clinical studies, is considered the best type of trial.^11^

As outlined above and based on the literature, this study presents the challenges encountered and solutions to overcome them during the conducting of an RCT (with crossover design). This study discussed the challenges of using RCTs and the chosen intervention, which was a multi-component training program (including computer-based components) in the study setting of Malaysia and with a study population of healthy older adults. To adhere to the study design, a style similar to the CONSORT statement was utilized to present the content of this paper. An overview of the study is illustrated in Table 1.

**Table 1:** Study outline.

Study stage	Description
Design	•RCT within-participants cross-over design including a multi-component intervention including strategy- and process-based (computer-based) components. •The study included two conditions: treatment/training and no-contact control conditions. •Ethical approval was sought from the University Putra Malaysia (UPM) ethics committee.
Sample	•40 potential participants were contacted, however only 25 were assessed for being eligible, and formally enrolled into the study based on the inclusion criteria: 1. subjects must be 55 years old and above, 2. educational level must be at least secondary level, and 3. show absence of; [i] history of neurological impairments (measured with MMSE (cut-off value=27) where scores for the sample ranged from 27 to 29 (M=27.68, SD=0.75), [ii] any major psychiatric disorder (taking any psychoactive medication (e.g., anti-depressive)) and learning disabilities, [iii] history of general anaesthesia, head traumas (in the last 6 months prior to the study), cerebrovascular disease, or neurological impairments, and [iv] drug/alcohol abuse. •Informed consents were collected from the participants.
Setting	•The sample was collected from the U3A Kuala Lumpur/Selangor active members, their family members and friends. •The intervention was conducted in the computer lab of the Faculty of Human Ecology, University Putra Malaysia (UPM).
Intervention	•Each intervention session lasted 2 hours. •There was 1 session per week for 6 weeks. •During each session, participants were exposed to intention implementation and other similar strategy-based exercises and a computer-based exercise (playing Virtual Week).
Data collection	•Baseline and post-intervention assessments for 4 outcome measures were assessed: PM (PRMQ and PM-Tasks*), IADL, GDS, and GAS
Data analysis	•Results of pre- and post-test assessments were reported as means (M), and standard deviations (SD). The General Linear Model (GLM) was used to show the effectiveness of the intervention.

* Prospective Memory-Tasks (PM-Tasks) is a computerized measurement tool developed to evaluate three types of PM tasks (event-based, time-based, and activity-based PM tasks). In the eventbased PM tasks section, a sequence of pictures including four shapes in each picture is presented at the center of the screen, and the participants are required to decide by pressing different keys for different shapes. If there is a bold and white shape in any sequence they are asked to press the spacebar (this is the PM task). In total, five bold and white shapes will be presented. In the time-based PM tasks section, everything is the same as the previous section except that a digital timer appears in the upper part of the screen. Each time the timer turns another full minute (e.g., 02:00, the last two digits are 00), participants are required to press the spacebar (PM task). Moreover, no bold and white shapes appear on the screen. The score ranges from 0 to 5 for this part. At the end of each part of the test, participants see a written phrase displayed on the screen and are asked to press “enter” upon seeing this phrase (activity-based PM task).

## METHODs

Randomization: study site, sample, and design

Selecting the right facility to conduct the study is a significant issue. To find the right sample it is important to consider the cooperation, availability, and flexibility of the facility staff. Facility staff tend to be reluctant to partake in these processes because they may assume their ongoing schedules will be interrupted or an additional work load added to their routine tasks. All participants were recruited from the University of the Third Age (U3A) Kuala Lumpur/Selangor. There were 19 women and 6 men in the sample. Participant age ranged from 55 to 74 years (M=63.32, SD=4.44) and years of education ranged from 10 to 20 years (M=14.69, SD=3.37). The U3A was chosen because of the pool of active and healthy members, and the cooperation and flexibility of the staff to arrange a smooth recruitment phase. Also, because the intervention included a computerized component, the participants’ level of education was a significant factor for choosing the U3A members as subjects for this study. The intervention was advertised by flyers distributed in the U3A classes among the students. Moreover, the invitation for participation was extended to include students’ family members and friends (due to the small number of active U3A members). The current study used the U3A, which is located at Malaysian Research Institute on Aging, University Putra Malaysia (UPM), as the recruiting facility. However, the intervention was conducted at the Faculty of Human Ecology, UPM. The researcher took responsibility for guiding the participants to the study location, thereby reducing the pressure on the U3A staff and increasing the retention rate (intervention success).

The recruitment phase for this study was a time-consuming stage. There were several challenges faced during this phase. First of all, the nature of the study created a challenge in recruiting potential participants. Conducting a training program with more than one session which is partially computer-based was a drawback from potential participants’ viewpoint. Furthermore, finding potential participants was a long process due to the fact that the U3A classes contained a small number of students, the same students partook in all courses, and their courses were not very frequent at the time of recruitment. And although the significance of the control group was explained, all potential participants stated if they took part, they would want to experience the training else they would not partake in the study.

Another issue was gaining the potential participants’ trust. As the study was mainly conducted by a foreign Ph.D. student, there were some cultural gaps and sensitivities regarding rapport development.

The next challenge was the duration of the screening session, which was 10-15 minutes. One major concern for the participants was travelling to the study site. Although it was explained to them that they would receive a voucher for their participation, most participants were reluctant to show up for the screening session. The duration of the assessment sessions was also a concern for the participants. On avereage, sessions were about 1.5-2 hours long, which was tiring for the participants.

Originally, the study design was set to be a randomized controlled trial (RCT) with parallel groups and due to the risk of participants’ attrition, a 25% drop-out was planned. However, as the number of the potential participants for the study was small, the design was changed to be a within-participants crossover trial to reduce the sample size required.^12^

### Administration

Availability of the participants is a significant factor when planning to conduct the intervention sessions, especially for memory training programs. The literature shows that time of day is a significant factor regarding the learning process and memory performance.^13^^,^ 14 Prior to conducting the intervention, the researchers considered all ongoing and future course schedules of the U3A in order to schedule the intervention sessions. However, most potential participants were reluctant to confirm their participation before the U3A had confirmed their course schedules for the following semester. As the participants were joining different courses at the U3A, their initial timetable posed a challenge to conduct all training sessions at the exact time of day.

### Transportation

Another significant factor to conduct interventions is the study location. Transportation can be a challenge, especially considering the seasonal climate, distance, and transportation method. The U3A is located in the Malaysian Research Institute on Aging, UPM. As a facility with at least 30 computers was required for each training session, and the Department could not provide this facility, the study location was moved to the Faculty of Human Ecology, UPM. The participants were not familiar with the study location and it was not easy for them to reach. The participants found it hard to get to some sessions on time because of the weather conditions where this could also affect their mood. Hence, transportation was problematic with regards to the consistency of the sessions and their duration.

### Treatment retention

Working with older adults and different cultures can affect the retention rate. In this study, some participants could not attend some sessions due to health issues, their role as a caregiver, and other family issues (e.g., a family member passing away). Having an extended family is part of normal culture in Malaysia, so participant attendance can be greatly affected by this matter.

### Treatment fidelity

Treatment fidelity is highly important due to its effects on reliability of the results. One of the main underlying treatment fidelity factors for this study was the way the intervention was conducted. Beside the standardized intervention procedure, which was conducted by the same researcher/facilitator for all participants, the facilitator’s behavior and attitude toward the participants was also important. The multi-component intervention was chosen carefully: a challenging, yet fun and real-life like computer game, Virtual Week, was used as the computer-based component of the intervention.^15^^,^ 16 The computerized version of the game (Virtual Week) was used in an adaptive manner. The number of tasks and level of difficulty were increased progressively. The number of PM tasks in Virtual Week was increased from 20 to 70. Moreover, to make participant performance more challenging, the clock was hidden from the main screen of the game. Also, the strategy-based training was conducted in a lively and engaging manner for the participants. Previous studies used implementation intentions to either improve a specific PM task (e.g., health tasks), or for other PM tasks and not for time-based PM tasks.^8^^,^ 9 The current study used the same strategy in a more personalized way for all PM tasks. This study used instructions for utilizing the strategy for each PM task and assigning homework to motivate the participants and maintain the training effects. All tasks and exercises were based upon the participants’ personal experiences and everyday tasks.

### Outcome measures

The primary outcome measure of this study was PM performance. Older adults tend to underestimate their own memory performance, so use of solely self-rated PM measurements might not show the true results. Conversely, using lab-based (e.g., computerized approaches) measurements alone may lead to results which are not necessarily accurate, because lab-based techniques are not naturalistic or similar to everyday tasks older adults may encounter.^17^ Hence, this study used two types of PM measures to evaluate PM performance among the participants: subjective/self-rated measurement (Prospective and Retrospective Memory Questionnaire (PRMQ)), and objective measurement (Prospective Memory Tasks (PM-Tasks)) which evaluated all types of PM (i.e., time-, event-, and activity-based PMs).

The secondary outcome measures of this study included the level of independence measured with Instrumental Activities of Daily Living (IADL), negative mood/depression measured with the Geriatric Depression Scale (GDS), and anxiety level assessed using the Geriatric Anxiety Scale (GAS). All measurements exhibited reliability and validity regarding their use in elderly populations. These measurements were chosen for their nature (being mainly self-report) to follow a more holistic and accurate assessment method.^17^ Moreover, such self-rating measurements can provide positive insight for the participants regarding their abilities and wellbeing. However, completing five different measurement instruments was time-consuming and tiring for the participants.

### Statistical analysis

The first step of handling data is to select the data analysis method. The data analysis method should clearly demonstrate the results and provide a better understanding of the findings. This study followed the guidelines of the CONSORT statement and chose “intention to treat” analysis to include all participants and avert any overestimated treatment effects. The second step of handling data is to determine how to handle missing data. Initially, this study chose to use the last value carried forward method for the missing data. However, there were no missing data.

## Results

### Lessons learned

All challenges faced while conducting this study could be classified into three categories. The challenges, and the solutions for them, are presented in the ensuing sections.

### Identifying and screening the eligible participants

Initially, the U3A members did not find the study very interesting (they were not motivated to play a computer game for 6 weeks). It is suggested to target a larger sample pool and ideally to choose computer-savvy subjects or run a short basic computer training course for potential subjects before conducting the actual intervention.

### Obtaining consent and trust

There were five main challenges to gaining trust: [i]researcher’s background: the researcher’s age, gender, and credibility were constantly questioned by the potential participants. They were concerned about the researcher’s gender; they were reluctant to partake if the researcher was male. The participants stated that female facilitators were more caring and patient. The intervention phase was originally planned to be conducted by a female researcher and this was therefore not a concern for the participants, [ii] the program: as mentioned above, most of the potential participants were not computer savvy and were also concerned regarding the efficacy of computerized programs. The potential participants were given information regarding the computer-based component and possible positive effects of it, [iii]fellow participants: participants were concerned about the other participants’ age, gender and race. The U3A Kuala Lumpur/Selangor had a predominantly ethnic Chinese member pool and therefore the concern regarding other participants was addressed to some extent, [iv] group allocation: most participants agreed to take part, but only if they received the training. The importance of the control group was explained and, moreover, participants were informed they would receive the training, and [v] lack of knowledge regarding age-related changes: the participants claimed they were too old to learn and their memory would probably get worse due to aging. They were informed and assured about cognitive age-related changes and techniques to prevent these changes or to promote their cognitive functions.

### Maintenance of participation

There was a 3-month gap between the recruitment and intervention phases because, initially, there were not enough participants recruited. Furthermore, participants expressed their concern regarding their free time. To encourage the participants to stay in the program, they were regularly contacted by the researchers and reminded about the program. Additionally, the participants were given some information regarding the potential positive effects of the program on their memory performance. Additionally, the intervention was planned for a day on which all participants could take part. Also, all training sessions were planned to be conducted at the same time of day. The only remaining issue was regarding the replacement of missed classes. However, all participants were encouraged to take part in the class and on the scheduled dates. If they had to miss a class, they were given some options to replace the class that they had missed. The possible issues to be considered for future studies are presented in Table 2.

**Table 2:** Possible issues to consider when conducting an RCT for PM training among healthy older adults.

Study design	Intervention administration	Retention and treatment fidelity
**Selecting the right facility** •Cooperation and flexibility •Activities timetable •Necessary equipment **The study budgets** •Number of participants •Number of research assistants needed •Remuneration **The recruitment process** •Duration/project timing •Flexibility **The screening process** •Inclusion and exclusion criteria •Screening tools •Duration of the session	**Time of day** **Duration and frequency of the sessions** **Participant schedule** **Transport** **Weather conditions**	**Standardized procedure** **Training the facilitators** **Measurement tools** •Self-reports •Insight/feedback •Reliability and validity **Facilitator characteristics** •Age •Gender •Nationality •Capability •Attitude **Fellow participants’ characteristics** •Age •Gender •Nationality •Attitude **Training** •Training type •Training components •Tolerability •Flexibility (replacement sessions)

## Discussion

Although PM is an essential part of everyday life, especially among older adults, PM training is often understudied in older adult populations. A few PM training programs have been conducted^9^ and the present study results are in line with findings of previous similar studies.^8^^,^ 10 ^,^ 18 ^,^ 19 Nevertheless, this study overcame the shortcomings of previous studies (e.g., lack of robust design, follow-ups, or good training methods) and extended their training programs by using a tailor-made training approach to include metacognitive factors affecting PM performance among older adults. This study used the training program to promote all types of daily living PM tasks and evaluated the training and transfer effects of the training program regarding the ability to live independently and psychological Well-being (i.e., levels of negative mood and anxiety). Moreover, this study used a more realistic approach to train PM (e.g., using Virtual Week and implementation intentions) with explicit instructions for participants and did not use a pure laboratory-based approach which could not be associated with real-life everyday living tasks. Hence, the current study found that the multi-component PM training had significant impact on PM performance, the ability to live independently, and psychological well-being among the healthy older adults.

This paper discussed some of the challenges faced when conducting an RCT involving a multi-component PM intervention. Although this study did not have a smooth recruitment phase, it had an excellent retention rate. The so-called ‘passive’ participation was made very ‘active’ due to the following factors: [i] the participants were given some information regarding age-related changes in a lively and participative manner with numerous personal examples from their own lives, [ii] not many technical terms were used to train them for the computer-based part of the intervention, [iii] all components of the intervention were utilized in a lively and empowering way to encourage progress among the participants, and [iv] patience and tolerance toward participant mistakes, empowering them to move forward, ensuring and encouraging them regarding their performance can make it easier for them to learn and later apply their knowledge. Personal traits of the facilitator can provide an empowering environment to engage the participants. As an example, there were some participants who missed a session and later mentioned that, because of the positive feedback and calls they received from the facilitator, they tried to arrange for a replacement session and wanted to improve even more. The participants stated that if they were not encouraged, they would feel nervous and demotivated to continue the exercises.

Overall, it is hoped that this study has provided some suggestions for future researchers who are planning similar RCTs, and supported the development of a suitable approach for dealing with these issues and challenges.
